# Evaluation of the Potential Effect of Transgenic Rice Expressing Cry1Ab on the Hematology and Enzyme Activity in Organs of Female Swiss Rats

**DOI:** 10.1371/journal.pone.0080424

**Published:** 2013-11-27

**Authors:** Yang Wang, Baoyang Wei, Yixing Tian, Zhi Wang, Yun Tian, Shuduan Tan, Shengzhang Dong, Qisheng Song

**Affiliations:** 1 College of Bioscience and Biotechnology, Hunan Agriculture University, Changsha, China; 2 Division of Plant Sciences, University of Missouri, Columbia, Missouri, United States of America; Instituto de Biotecnología, Universidad Nacional Autónoma de México, Mexico

## Abstract

To assess the safety of transgenic rice expressing Cry1Ab protein to vertebrates, the effect of Cry1Ab rice on broad health indicators in blood and various organs of Swiss rats were analyzed. The 30 and 90 day safety studies of Cry1Ab rice on female Swiss rats revealed that Cry1Ab rice had no significant effect on the several elements of blood lymph including hemogram, calcium ion concentration and apoptosis rate of lymphocytes, indicating that Cry1Ab protein could not affect the blood lymph of Swiss rat. Similarly, Cry1Ab rice had no effect on enzyme activities in a variety of organs of Swiss rat. However, Cry1Ab rice did have significant effects on the blood biochemistry indexes including urea, triglyceride (TG), glutamic oxalacetic transaminase (AST) and alkaline phosphatase (ALP) after the rats were fed with Cry1Ab rice for 30 days, but not after 90 days, indicating that Cry1Ab protein may influence blood metabolism for a short duration. Quantitative real-time PCR (qPCR) analysis of the 6 genes encoding enzymes responsible for the major detoxification functions of liver revealed that Cry1Ab rice exerted no influences on the levels of these transcripts in liver of Swiss rat, indicating that significant differences registered in part of the blood biochemical parameters in the 30 day study might result from other untested organs or tissues in response to the stress of exogenous Cry1Ab protein. The results suggest that Cry1Ab protein has no significant long-term (90 day) effects on female Swiss rat.

## Introduction

The widespread abuse of broad spectrum chemical insecticides in rice fields has resulted in environmental contamination, ecosystem deterioration and outbreak of pest populations, especially planthoppers in Asian countries [1]. The development and commercialization of transgenic rice expressing insecticidal protein such as *Bacillus thuringiensis* (*Bt*) toxin could alleviate this hazard problem. Thus a variety of transgenic rice lines carrying a single *Bt* gene expressing the insecticidal proteins, namely Cry1Aa [2], Cry1Ab [3], Cry1Ac [4], Cry1B [2], Cry1C* [5], Cry1Ca1 [6], Cry2A [7] and Cry9C [8], have been developed to control rice pests. A *Bt* rice cultivar (Huahui 1) and its hybrid line (Shanyou 63) has recently been approved for a limited commercialization trial in Hubei Province, China for a 5-year period (2009–2014) [9].

Cry1Ab protein is specifically toxic to lepidopterans. The transgenic rice expressing Cry1Ab protein such as Shanyou 63 inhibits growth and development of lepidopteran pests. Although transgenic rice expressing Cry1Ab protein can bring huge benefits, its potential risks have drawn broad attention. Studies showed that Cry1Ab protein expressed in transgenic rice could accumulate not only in target pests but also in non-target insects via food chain. Some reports regarding non-target effects of *Bt* rice on insects were related to planthoppers, specifically concerning effects on the feeding and oviposition behaviors [10], [11] or field population dynamics [12], [13], [14] of planthoppers between *Bt* rice and their non-*Bt* parental rice, as well as the presence of the *Bt* toxin in planthoppers [15], [16], [17]. Chen et al. (2009) showed Cry1Ab protein expressed by transgenic rice could be transferred to the pirate otter-spider *Pirala piraticus* through its prey the brown planthopper (BPH) [18].

To assess the safety of Cry1Ab protein to vertebrates, toxicological evaluation of transgenic rice (KMD1) expressing Cry1Ab protein on Sprague-Dawley rats was performed and no adverse effects of Cry1Ab rice on rats were observed in terms of animal behavior, weigh gain and feed utilization rate [19]. Schroder et al. (2007) conducted a 90-day safety study of genetically modified rice expressing Cry1Ab protein in Wistar rats and no adverse effects on animal behavior, weight gain and standard hematological and biochemical parameters were observed [20]. Although no adverse effects of Cry1Ab protein on Wistar rats in an immunotoxicological study were detected when the rats were fed with Cry1Ab rice for 28 or 90 days, Cry1Ab protein were capable of inducing an antigen-specific antibody response [21]. Mesnage et al. (2013) tested for the very first time Cry1Ab and Cry1Ac *Bt* toxins on the human embryonic kidney cell line 293 on three cell death biomarkers: measurements of mitochondrial succinate dehydrogenase, adenylate kinase release by membrane alterations and caspase 3/7 inductions [22]. They found that Cry1Ab caused cell death from 100 ppm while Cry1Ac did not. The Roundup is necrotic and apoptotic from 50 ppm. When combined in use, Cry1Ab and Cry1Ac reduced caspases 3/7 activations induced by Roundup. They argue that modified *Bt* toxins are not inert on nontarget human cells, and that they can present combined side effects with other residues of pesticides specific to GM plants. Therefore, additional insight into possible physiological and biochemical alterations in response to feeding Cry1Ab rice to rats is needed to provide data relevant to safety of transgenic rice to vertebrates.

In this study, we chose Swiss rat as a model system to test the potential effects of Cry1Ab rice on the hematology, organ relative weight and enzyme activities in a variety of organs, including the hemogram, calcium ion concentration, apoptosis rate of the blood lymphocytes, blood biochemistry and enzyme activities including catalase (CAT), acetylcholine esterase (AChE), superoxide dismutase (SOD) and glutathione (GSH) in heart, liver, spleen, brain, kidney and ovary. Then we used quantitative real-time PCR (qPCR) to analyze the transcript levels of 6 genes encoding for the major detoxification enzymes in liver. The results could be used as a scientific basis for the safety evaluation of genetically modified rice expressing Cry1Ab protein.

## Materials and Methods

### Rice sample preparation

The seeds of the transgenic Shanyou 63 rice expressing Cry1Ab protein and its non-transgenic parental wild type Shanyou 63 rice were obtained from the Life Science College, Hunan Normal University. The Cry1Ab expressing rice and control rice were grown under nylon net to exclude insects and predators from neighboring fields and managed separately in the experimental farmland, Hunan Agricultural University. No insecticides were used in the field during the entire experiment period. The rice grain was harvested, dried and stored at free of mycotoxin contamination condition in glass desiccators at room temperature. Cry1Ab content in the transgenic rice grain was quantified using a *Bt* Cry1Ab/Ac ELISA reagent box (American EnviroLogix Corporation). The *Bt* rice contained 0.122 ng/g Cry1Ab protein while control rice had no detectable Cry1Ab protein. The *Bt* and control rice was then used to feed the Swiss rice as described below.

### Swiss rat sample preparation and ethics statement

Forty mature and pathogen free (SPF) Swiss rats were ordered from the Hunan SJA Laboratory Animal Co.,Ltd. The rats in test group were fed with 6 g unhulled *Bt* rice per day and those in control group with 6 g unhulled non-*Bt* rice under the room temperature for either 30 or 90 days. Swiss rats were weighted individually before and after dissection. All experiments were performed according to the Hunan Community Rules of Animal Care with the permission number 38–61 of Hunan Agricultural University Veterinary Services (China). All experiments were covered by agreement No.2012-VIDH-065 (September 13^th^, 2012) from the Veterinary Inspection Department of Hunan Agricultural University (China).

### Blood and organ sample preparation

Blood was collected individually from eyeball veins of rats after being fed for 30 and 90 days in a heparin anticoagulation tube for hemogram and blood lymphocyte tests, and the brain, kidney, liver, spleen and marrow were dissected respectively for enzyme activity assays. The blood and organ samples were immediately processed according to the method of Xu [23].

### Potential effect of Cry1Ab rice on blood lymphocyte functions, organ weight and Enzyme activities in organs

The apoptosis rate and calcium ion level in the blood lymphocytes of Swiss rat fed with *Bt* or non-*Bt* (control) rice for 30 and 90 days were tested using the FCM FP1000 Flow Cytometry System (Beckman Corporation). The blood biochemistry indexes were tested using the OLYMPUS AU400 automatic biochemical analyzer. Each of 10 female Swiss rats was tested separately in this study. After dissecting the rats, we weighed the organs including heart, liver, spleen, brain, kidney and ovary. Then we cut off 0.2 g tissue from each of these organs including brain, kidney, liver, spleen and marrow to assay the activities of enzymes including AchE, CAT, SOD and GSH. The AchE enzyme activity was tested using the method of Gorun [24]. The CAT, GSH-Px and SOD enzyme activities were tested using the enzyme activity reagent box purchased from Chinese Nanjing Jiancheng Bioengineering Institute.

### Quantitative real-time PCR (qPCR) analysis of enzyme genes in liver

The primers of 6 genes encoding AchE, CAT, SOD (isoform 1, 2 and 3), alkaline phosphatase (ALP), glutamic oxalacetic transaminase (AST) and alanine aminotransferase (ALT) were designed from the conserved sequences of each gene in Genbank with Primer 3.0 software and synthesized by Shanghai Sangon Biological Engineering Technology & Service Co. Ltd (Table 1).

**Table 1:** Primers used for Real-Time PCR analysis of indicated enzyme transcripts in liver cells.

Primer	Sequence(5'->3')		Product size(bp)	Annealing temperature(°C)
Ache	Forward	gtggacaccataccccagac	163	58
	Reverse	ggttcccactcggtagttca		
Cat	Forward	cctcgttcaggatgtggttt	220	59
	Reverse	agctgagcctgactctccag		
Sod1	Forward	ccagtgcaggacctcatttt	197	59
	Reverse	ttgtttctcatggaccacca		
Sod2	Forward	ccgaggagaagtaccacgag	174	59
	Reverse	gcttgatagcctccagcaac		
Sod3	Forward	tctgcagggtacaaccatca	226	60
	Reverse	acctccatcgggttgtagtg		
Alpl	Forward	gctgatcattcccacgtttt	204	60
	Reverse	ctgggcctggtagttgttgt		
Got1l1	Forward	atgtacccacagcccagaag	197	59
	Reverse	caccaggggcaagtactcat		
Gpt	Forward	aaggctaaactcacggagca	174	58
	Reverse	ctcttccaggaggcacagac		
GAPDH(MUS)	Forward	aactttggcattgtggaagg	132	58/59/60
	Reverse	ggatgcagggatgatgttct		

Total RNA was extracted from the liver tissues of female Swiss rats after being fed with *Bt* or non-*Bt* rice for 30 and 90 days using the Trizol reagent (Invitrogen Life Technologies, China) following the manufacturer’s protocol. The RNA quantity was examined using a spectrometer at A260 and RNA quality was checked with the ratio of A260/A280 and verified by agar gel electrophoresis. First-strand cDNA was synthesized from 2 µg DNAse-treated total RNA using an oligo-dT18 primer and RevertAidTM M-MLV reverse transcriptase. qPCR amplification and analysis were carried out on an Applied Biosystems (ABI) 7900 Real-Time PCR System. The final reaction volume was 25 µl using ABI SYBR green Supermix (ABI). The PCR program was: hold at 94°C for 3 min and then at 94°C for 30 seconds, 59°C for 30 seconds and 72°C for 45 seconds, repeating 40 cycles with a final extension at 72°C for 5 min. Melting curve analysis was applied to all reactions to ensure homogeneity of the reaction product. The relative abundance of transcript was normalized and calculated against that of the GAPDH gene as an endogenous reference using the 2^−ΔΔ^C_T_ method [25].

### Data analysis

Significant differences were using the SPSS 19.0 version of Wilcoxon signed-rank test. Significant difference was set at *p*<0.05 and designated with *.

## Results

### Effects of Cry1Ab rice on hematological indicators

After the rats were fed with the *Bt* or non-*Bt* rice grains for 30 and 90 days, the blood was drown for hematological analysis. From Table 2, no significant differences in all 18 categories of hematological indicators between the test and the control groups were observed.

### Effect of Cry1Ab rice on the apoptosis rate of blood lymphocytes

**Figure 1:**
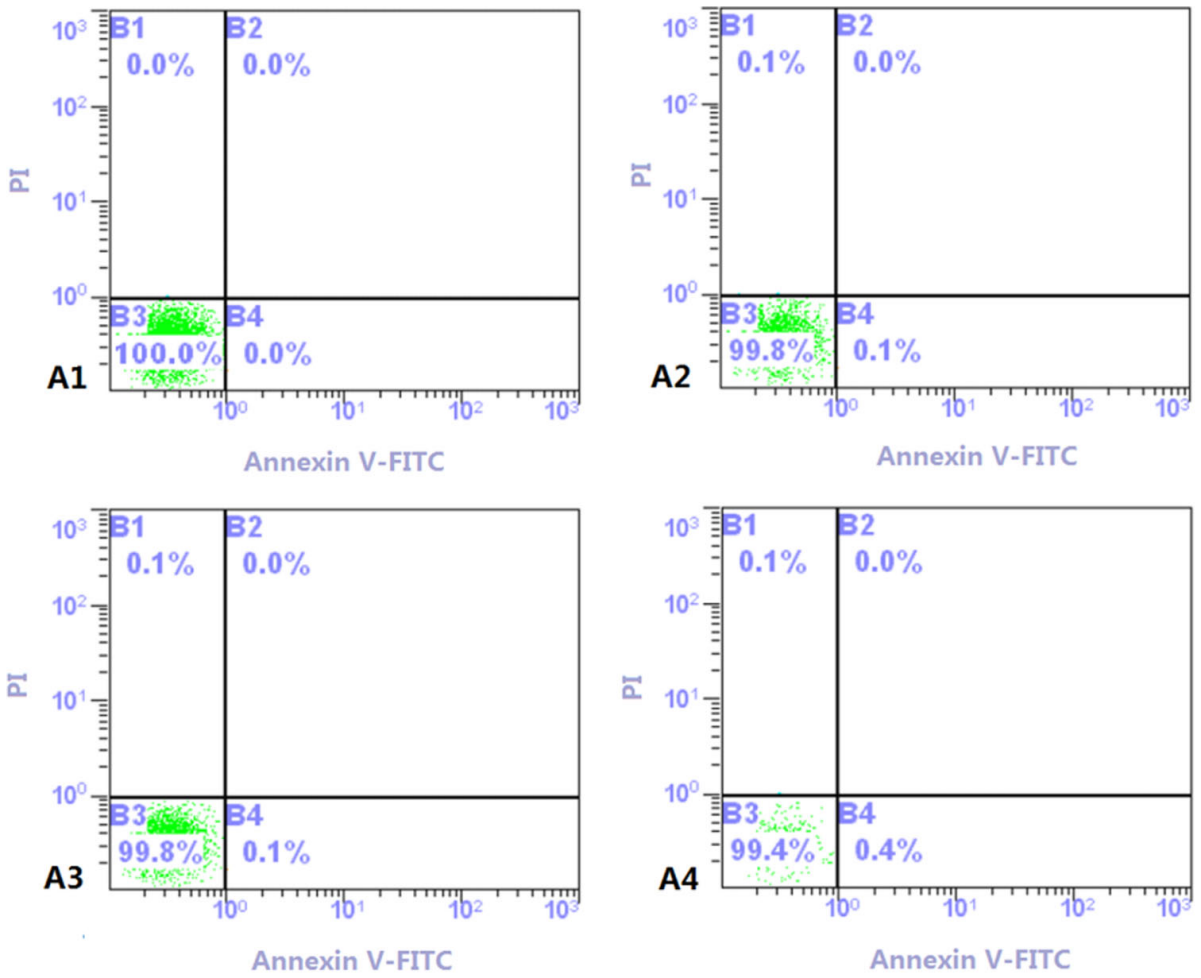
The variation of apoptosis rate in Swiss rats under the effect of Cry1Ab protein. A1: the 30 day feeding Swiss rats in control group. A2: the 90 day feeding Swiss rats in control group. A3: the 30 day feeding Swiss rats in test group. A4: the 90 days feeding Swiss rats in test group. Note: The X coordinate means the percent of dyeing Annexin V cells. The Y coordinate means the percent of dyeing PI cells. The B1 coordinate means the percent of necrosis cells and the late apoptosis cells. The B2 coordinate means the percent of dead cells of mechanical damage. The B3 coordinate means the percent of survived cells. The B4 coordinate means the percent of the early apoptosis cells.

Cry1Ab protein had no significant effects on the apoptosis rate of blood lymphocytes of Swiss rat in both 30 and 90 day studies (Fig. 1). The number of apoptosis cells in the 30 days study attained near 0%. Although in the 90 days study, the number of apoptosis cells was 0.1% in the control group and 0.4% in the test group, the differences between them had not attained the significant level.

### Effect of Cry1Ab rice on the calcium ion content of blood lymphocytes

**Figure 2:**
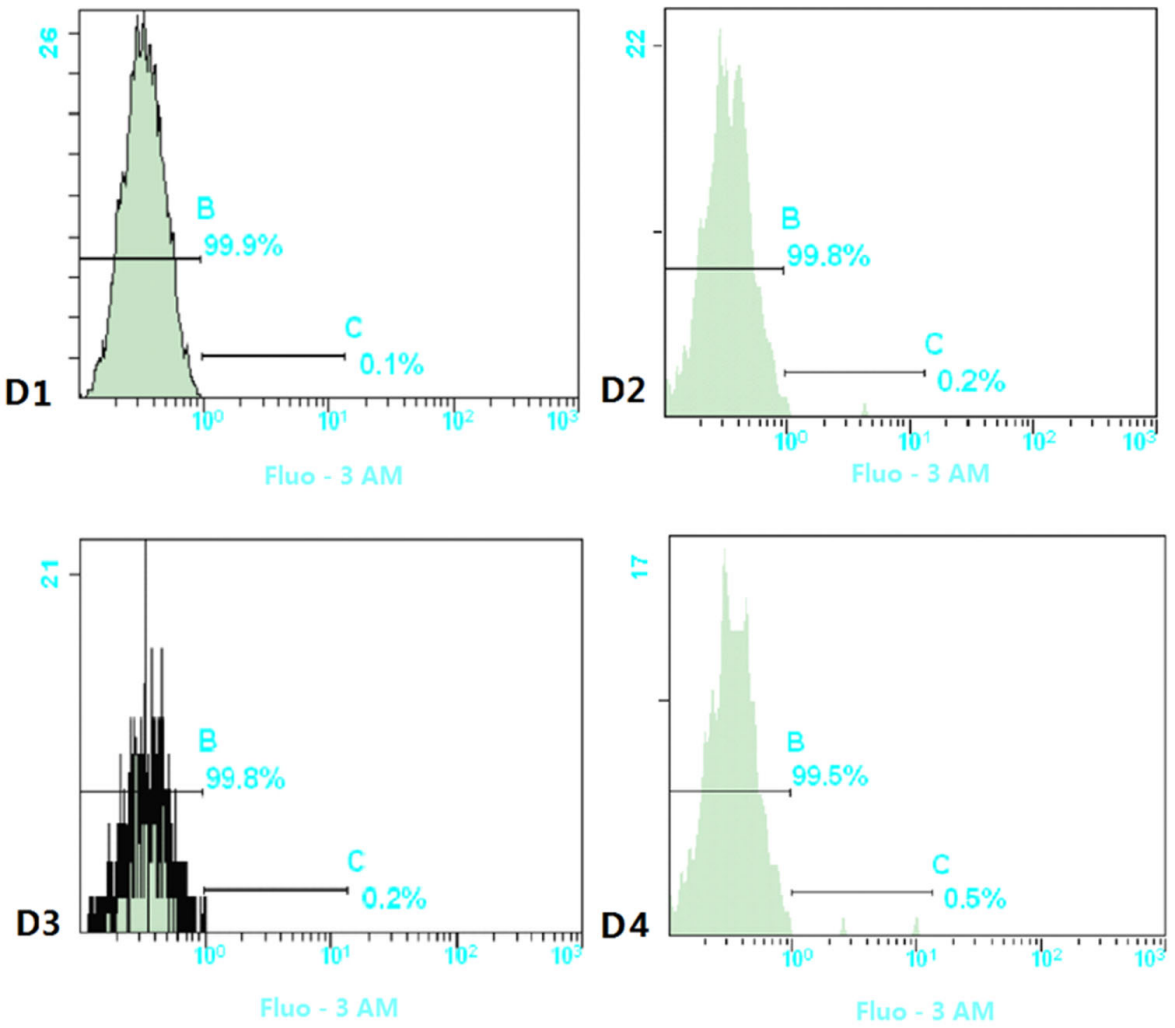
The variation of calcium ion content in blood lymphocytes of rat under the effect of Cry1Ab protein. D1:the 30 days feeding Swiss rats in control group. D2: the 90 day feeding Swiss rats in control group. D3: the 30 day feeding Swiss rats in test group. D4: the 90 day feeding Swiss rats in test group.

As shown in Fig. 2, calcium ion content in the blood lymphocytes of the test group was higher than that in the control group. However, the differences had not reached the statistically significant levels, indicating that no effect on the blood lymphocytes is apparent.

### Effect of Cry1Ab rice on blood biochemistry

**Figure 3:**
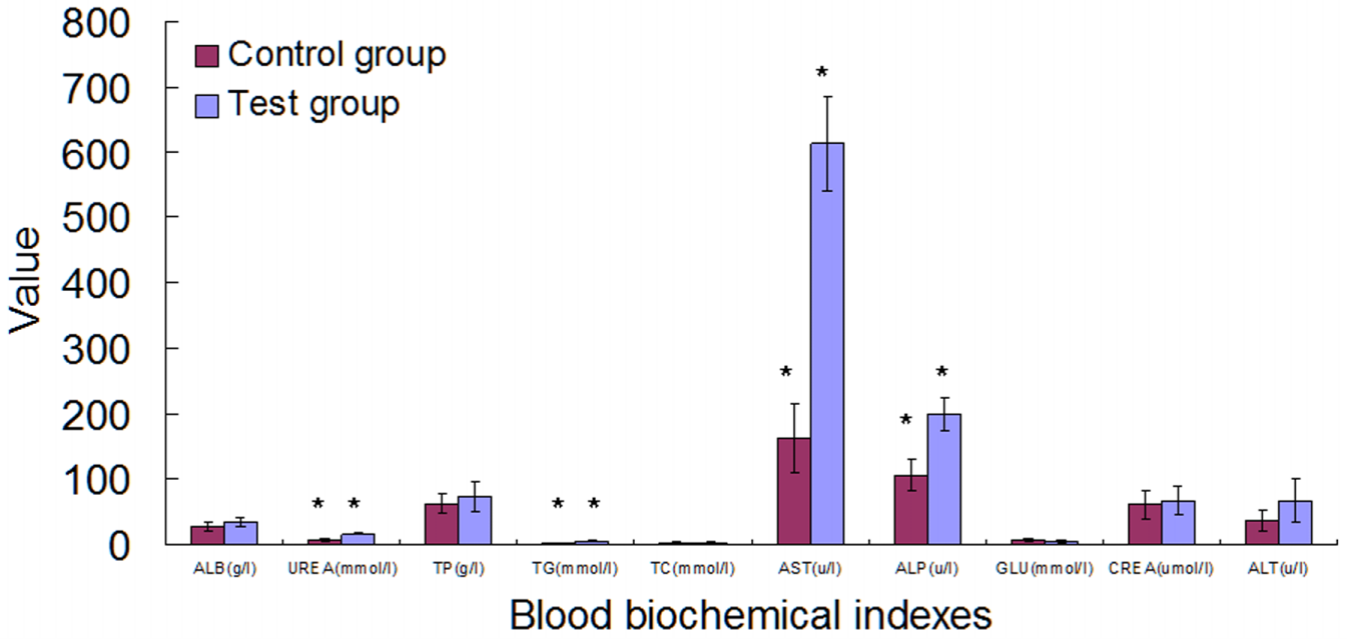
Effect of Cry1Ab protein on the blood biochemistry in Swiss rat that feeding for 30 days. The blood biochemistry indexes including ALB, UREA, TP, TG, TC, AST, ALP, GLU, CREA and ALT were assayed. The red bar represents the control group and the blue bar represents the test group.

**Figure 4:**
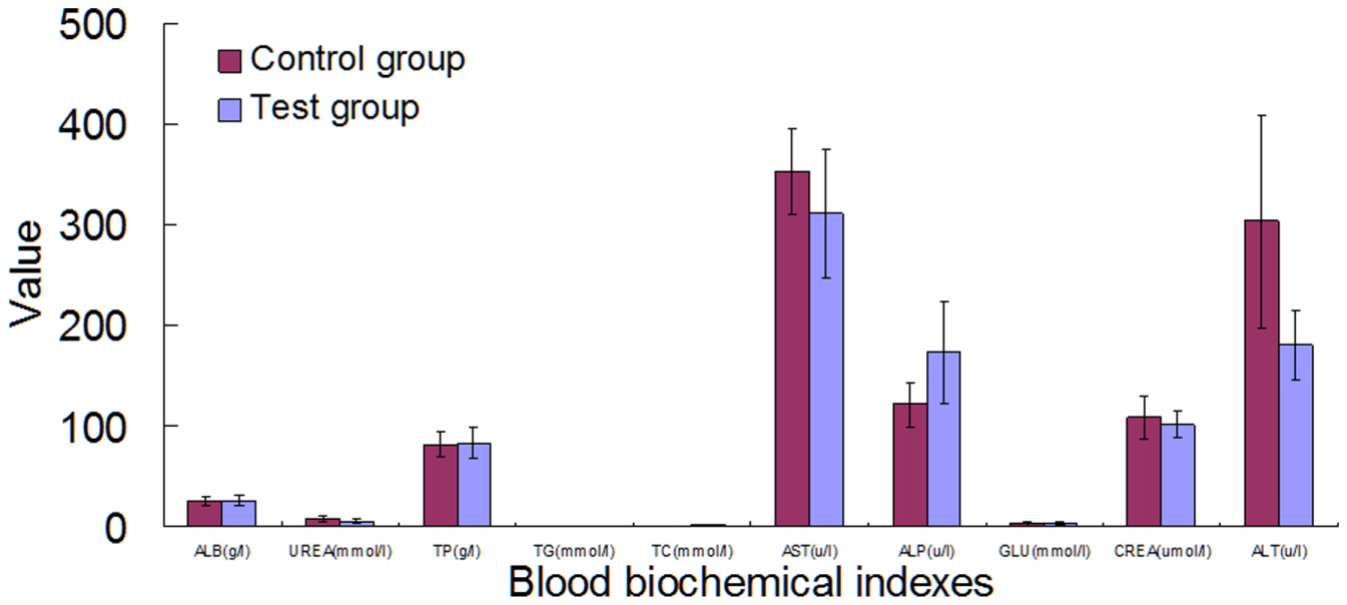
Effect of Cry1Ab protein on the blood biochemistry in Swiss rat that feeding for 90 days. The blood biochemistry indexes including ALB, UREA, TP, TG, TC, AST, ALP, GLU, CREA and ALT were assayed. The red bar represents the control group and the blue bar represents the test group.

The 30 day study shows that some blood biochemical factors including urea, triglyceride (TG), AST and ALP were significantly higher in the treated group than that in the control (*p*<0.05) (Fig. 3). However, no significant differences existed in all of the blood biochemical factors between the test and control groups in the 90 day study (Fig. 4).

### Effect of Cry1Ab rice on organ relative weight

**Figure 5:**
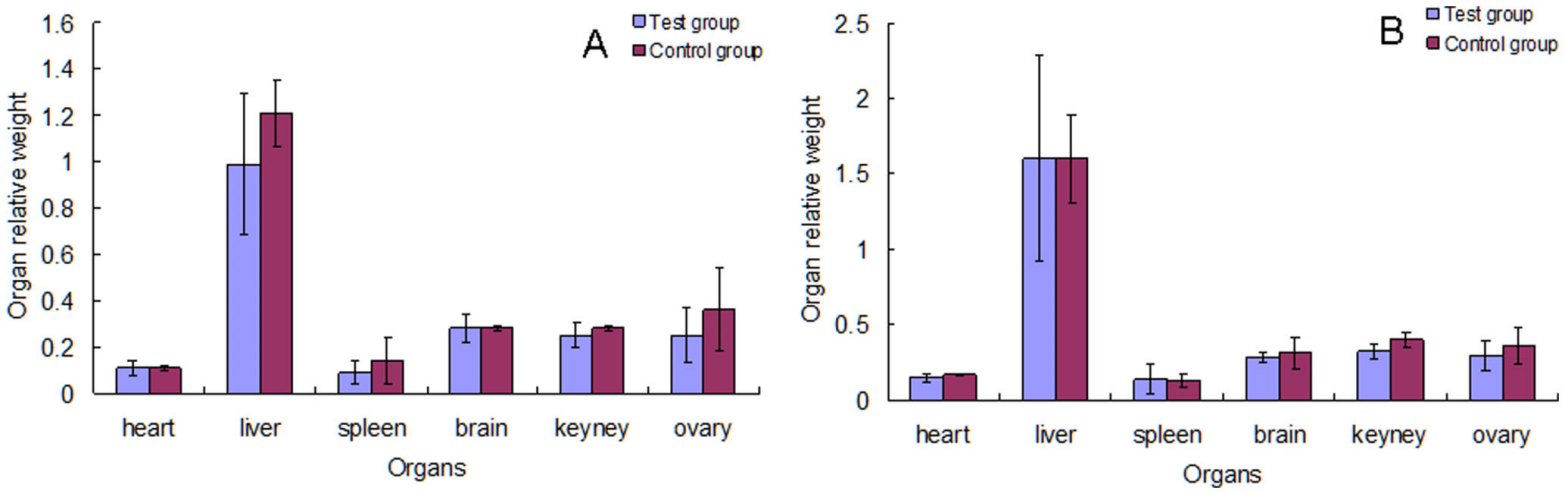
Effect of Cry1Ab protein on the organ relative weight of Swiss rat. The organ relative weight was calculated by organ weight/body weight. The body weight of rat was weighed before the rat was dissected and the organ weight of rat was weighed after the rat dissection. A: the organ relative weight in Swiss rat that feeding for 30 days. B: the organ relative weight in Swiss rat that feeding for 90 days.

There were no significant differences in organ weight between the test and control groups in the 30 and 90 day studies (Fig. 5A and B), indicating that the Cry1Ab protein has no effect on the organ development in female Swiss rats.

### Effect of Cry1Ab protein on the enzyme activities of organs

**Figure 6:**
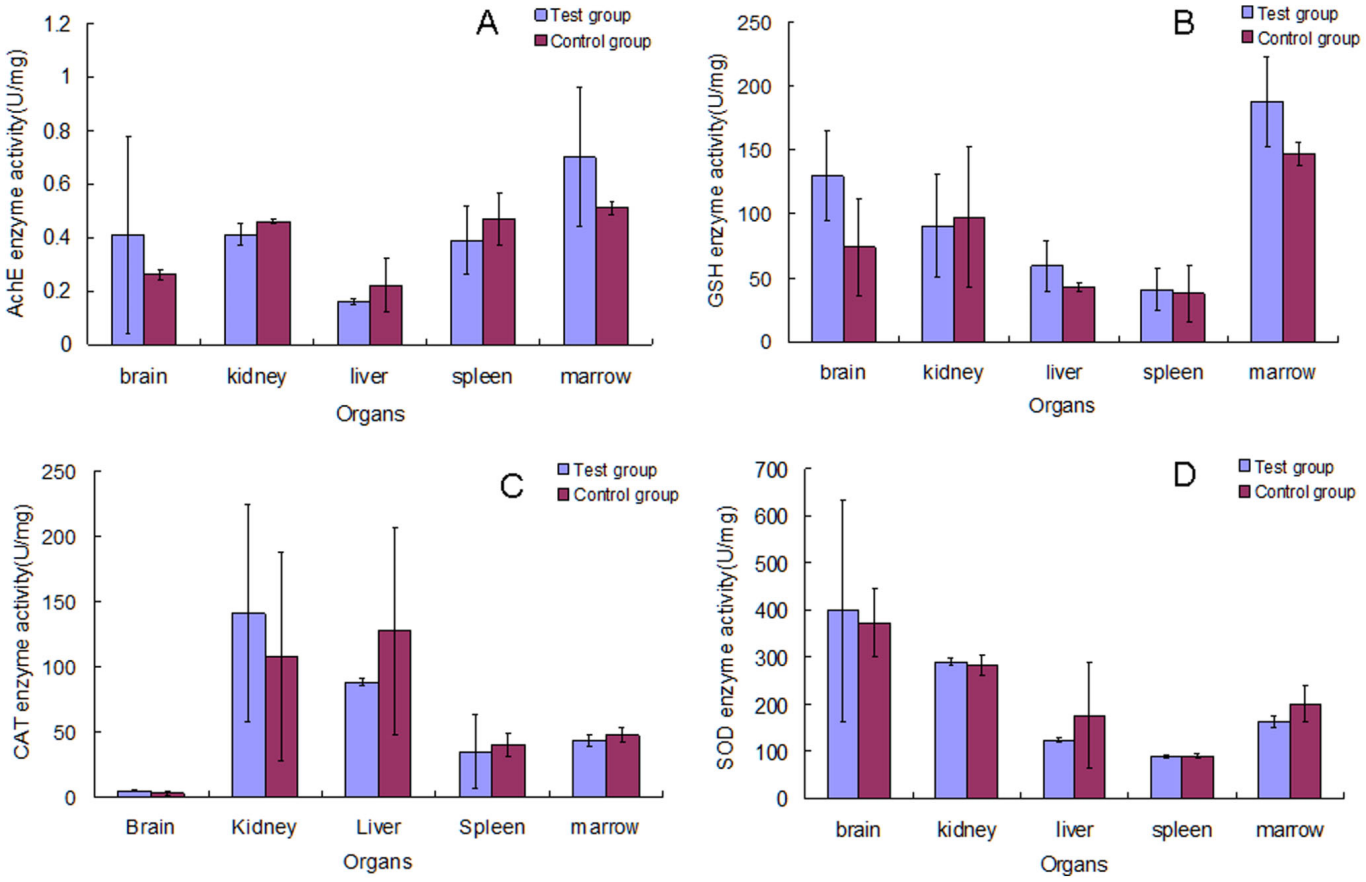
Effect of Cry1Ab protein on the enzyme activity of organs in Swiss rat that feeding for 30 days. The enzyme activities of the organs including brain, kidney, liver, spleen and marrow were assayed. A: the AchE enzyme activity. B: the GSH enzyme activity. C: the CAT enzyme activity. D: the SOD enzyme activity.

**Figure 7:**
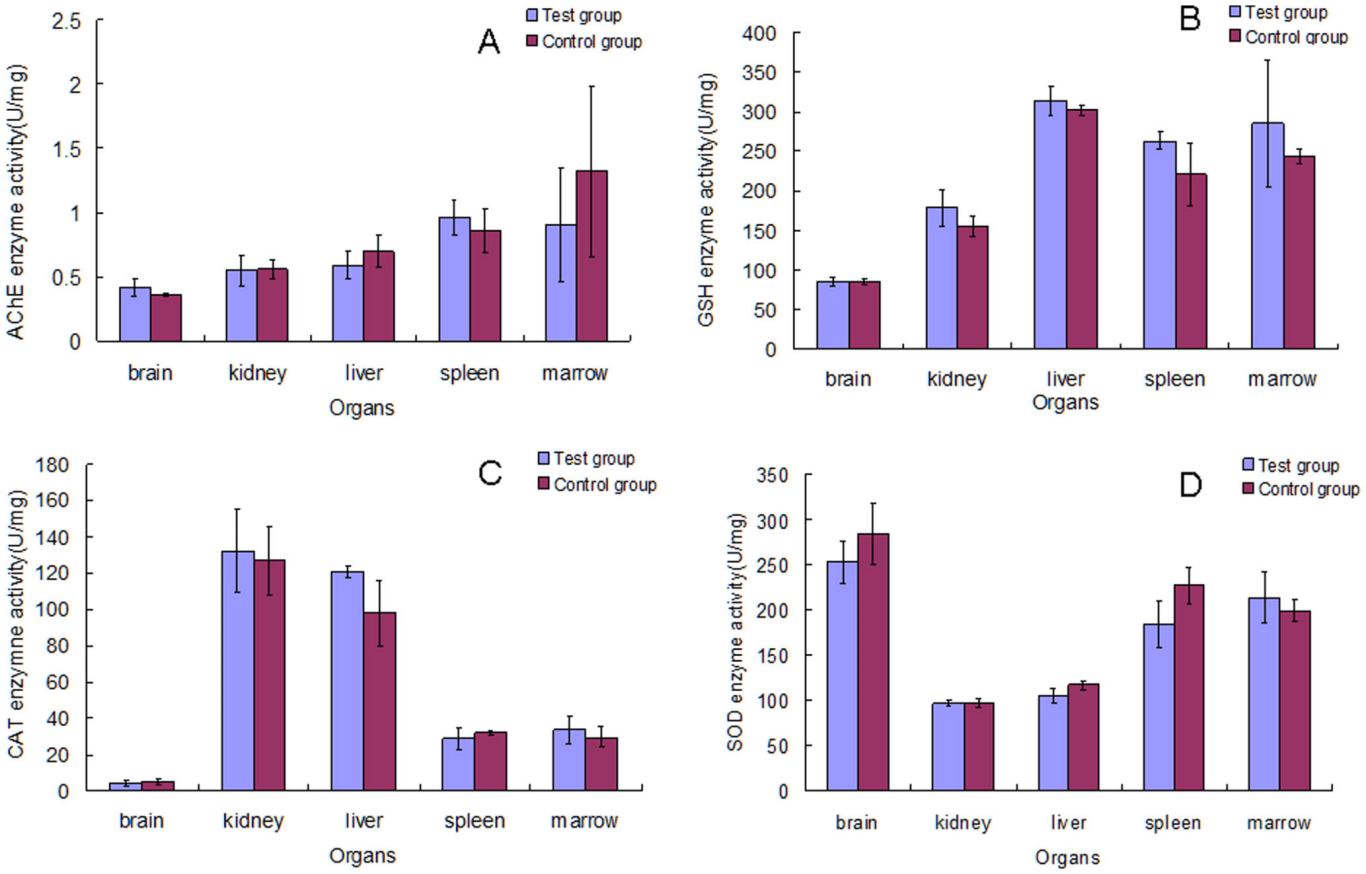
Effect of Cry1Ab protein on the enzyme activity of organs in Swiss rat that feeding for 90 days. The enzyme activity of these organs including brain, kidney, liver, spleen and marrow was assayed. A described the AchE enzyme activity. B described the GSH enzyme activity. C described the CAT enzyme activity. D described the SOD enzyme activity.

As shown in Fig. 6 and Fig. 7, there were no significant differences in AchE, CAT, SOD and GSH activities between the test and control groups in both 30 and 90 day studies. This indicates that Cry1Ab protein has no significant effects on the enzyme activities in organs of female Swiss rats.

### qPCR analysis of transcript levels of enzymes in liver

**Figure 8:**
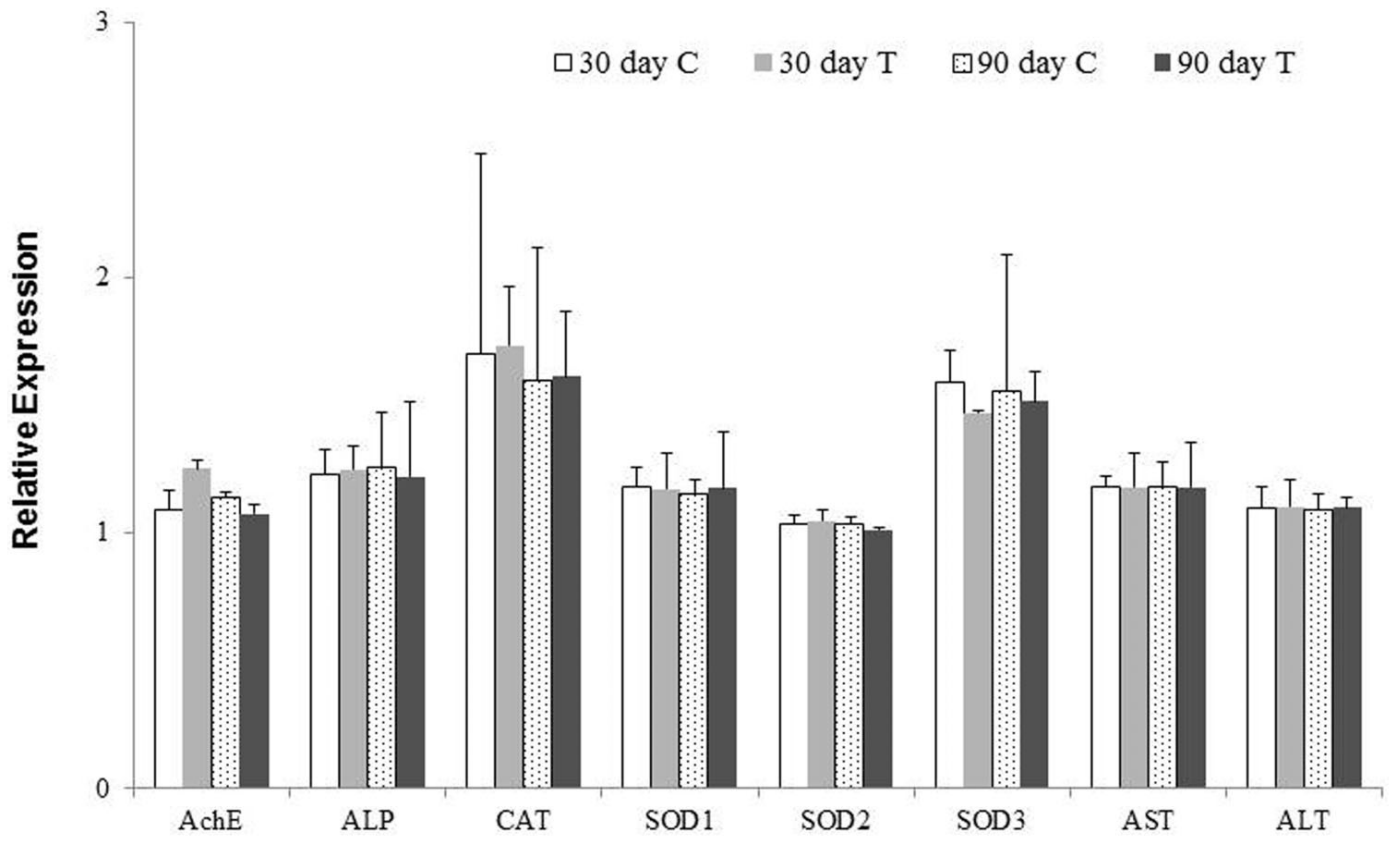
qPCR analysis of several genes in Swiss rat fed with Cry1Ab protein for 30 and 90 days. C, control; T, fed with Cry1Ab (test). Columns represent means of three individual measurements and bars indicate standard errors of the mean (SEM). The genes encode AchE, CAT, SOD1, SOD2, SOD3, ALP, AST and ALT respectively.

The qPCR results revealed that there were no effects of Cry1Ab protein on transcript levels of these 6 genes encoding AchE, ALP, CAT, SOD (isoform 1–3), AST and ALP (Fig. 8), results consistent with the enzyme activity assays in liver (Fig. 5, 6, 7).

## Discussion

The variation of physical signs in blood reflects the health condition of animals [26]. The apoptosis rate and calcium ion content of blood lymphocytes also reflect the immune system condition of Swiss rats. This study showed that no effects of Cry1Ab protein exists on the hemogram (Table 2), calcium ion concentration (Fig. 1) and apoptosis rate of the blood lymphocytes (Fig. 2), a result consistent with the previous report on hematological parameters in Wistar rat by Schroder et al. (2007) [20], indicating that Cry1Ab could not affect the hematological health of Swiss rat. However the results of blood biochemistry show that some biochemistry indexes in the test group, including urea, TG, AST and ALP were significantly higher than that in the control group in the 30 day study (Fig. 3) while the abnormality disappeared in the 90 day study.

**Table 2:** Effects of transgenic *Bt* rice on the hematological indicators of rats.

	30 day study		90 day study	
	Control group	Test group	Control group	Test group
WBC (10^9^/l)	18.90±6.25	21.47±2.90	17.93±6.91	15.80±6.46
Lymph # (10^9^/l)	9.37±3.79	10.73±1.46	11.23±6.07	8.73±3.85
Mon # (10^9^/l)	1.50±0.56	1.83±0.48	1.13±0.51	1.27±0.32
Gran # (10^9^/l)	8.03±2.25	8.90±1.48	5.57±2.11	5.80±2.33
Lymph (%)	48.33±7.31	49.97±3.72	59.47±16.14	54.57±2.31
Mon (%)	8.17±1.02	8.47±1.19	7.00±3.48	8.50±1.40
Gran (%)	43.50±6.47	41.57±3.98	33.53±12.67	36.93±1.50
RBC (10^12^/l)	9.72±0.47	9.51±0.59	9.32±1.09	8.90±1.08
HGB (g/l)	189.00±1.73	184.67±17.39	181.00±22.87	156.67±21.22
HCT9 (%)	44.43±0.65	44.90±2.98	43.73±4.71	39.33±5.30
MCV (fL)	45.80±1.61	47.30±2.43	47.07±2.43	44.20±0.60
MCH (pg)	19.43±0.85	19.33±1.29	19.40±0.87	17.57±0.42
MCHC (g/l)	425.00±3.46	401.00±27.71	412.67±9.61	397.67±9.71
RDW (%)	13.70±1.20	16.03±2.28	14.10±2.60	17.30±4.65
PLT (10^9^/l)	371.00±237.31	325.00±84.48	446.33±304.00	256.66±110.74
MPV (fL)	5.63±0.12	5.63±0.21	6.17±0.38	5.73±0.25
PDW	16.87±0.60	16.63±0.55	17.10±0.46	16.50±0.44
PCT (%)	0.21±0.14	0.18±0.05	0.27±0.18	0.15±0.07

The contents of ALB, UREA, TP, TG, TC, AST, ALP, GLU, CREA and ALT in blood have firmly relations with the liver function. The increased UREA and TG levels and elevated AST and ALP enzyme activities in the 30 days study, but not in the 90 day study, indicate clearly that the effects of Cry1Ab protein on these parameters last only for a short duration. To investigate whether the short term effect on the above mentioned biochemistry indexes observed in the 30 day study was due to alternation in liver function, qPCR analysis of the genes encoding 6 major detoxification enzymes in liver were performed. qPCR results revealed that the Cry1Ab protein did not influence the transcript levels of these enzymes (Fig. 8), a result consistent with what registered in liver enzyme activity assays although only 4 enzyme activities were assayed (Fig. 5, 6, 7), suggesting that the significant differences in part of the blood biochemical parameters in the 30 day study may not result from liver or other organs tested. Further investigation is needed to identify the sources of the short duration effect on these blood biochemical indexes, such as intestine, lymphocytes, etc. Nevertheless, with the body adapts to the Cry1Ab toxin in 90 days, the abnormal indexes revert to the normal levels, indicating that there are no adverse long term effects of Cry1Ab on blood biochemical indexes. Recent reports revealed that Cry1Ab gene and protein can be detected in the gastrointestinal digesta, but not in the kidneys, liver, spleen, muscle, mesenteric lympho node, heart or blood of pigs and calves fed with Cry1Ab protein [27], [28], [29]. Interestingly, a trace among of Cry1Ab protein could also be detected in pregnant women, their fetuses and non-pregnant women, presumably being exposed to *Bt* contamination in environment [30]. Whether the presence of Cry1Ab protein in the intestines of pigs and calves or in human blood has any adverse effect on animals and human needs further investigation. Walsh et al. (2012) show in their study that lymphocyte counts are higher (P<0.05) in pigs fed Cry1Ab/isogenic than pigs fed Cry1Ab or isogenic on day 100 while erythrocyte counts are lower in pigs fed Cry1Ab or isogenic/Cry1Ab than pigs fed Cry1Ab/isogenic (P<0.05), but perturbations in peripheral immune response are not indicative of Th 2 type allergenic or Th 1 type inflammatory responses [31]. Kroghsbo et al. (2008) report that Cry1Ab is capable of inducing an antigen-specific antibody response when Wistar rats are fed transgenic rice expressing Cry1Ab [21]. These studies clearly indicate that intestine and/or lymphocytes might be the targets of interest for evaluation of the safety of *Bt*. The elevated UREA and TG levels along with increased AST and ALP enzyme activities in the 30 days study detected in the present study could result from intestine or other untested organs and tissues. Thus further investigation is needed to identify the source of these elevated indexes.

With the *Bt* rice feeding for 90 day study, we found that the organ relative weight of Swiss rats has no significant differences between the test and control groups. This phenomenon suggests that the organ development of the Swiss mice cannot be influenced by taking in the *Bt* unhulled rice for a long period of time. This result is consistent with previous studies showing that Cry1Ab has no effect on weight gain of Sprague-Dawley rat [19] and Wistar rat [20].

The study of Chubb shows that AchE that involved in cell development and maturation with the ability to promote neuronal development and nerve regeneration has the activities of carboxypeptidase and aminopeptidase [32]. Our study shows that the AchE enzyme activity in rat organs in the test group after feeding with Cry1Ab protein has no significant differences compared to that in the control group. These results verified that neuronal development and nerve regeneration of the rat could not be affected by Cry1Ab protein. There is a balance between generation and elimination of free radicals existing in the physiological process of all aerobic organisms [33]. The GSH-Px enzyme, CAT and SOD presented widely in aerobic organisms have functions of eliminating the free radicals. The present study shows that these three enzyme activities in the test group have no significant changes compared to the control group in the 30 and 90 day studies. The results of enzyme assay also show that the Cry1Ab protein taken in by Swiss rats cannot adversely disorder the enzyme system to influence the health of the rats.

The present study cannot exclude the possibility that the Cry1Ab protein may have effects on the other untested factors in hematology and organs in Swiss rat. Further research is needed to provide a comprehensive scientific basis for the safety evaluation of *Bt* crops.
